# Is there still a place for the cemented titanium femoral stem? 10,108 cases from the Norwegian Arthroplasty Register

**DOI:** 10.3109/17453674.2011.645194

**Published:** 2012-02-08

**Authors:** Geir Hallan, Birgitte Espehaug, Ove Furnes, Helge Wangen, Paul J Høl, Peter Ellison, Leif I Havelin

**Affiliations:** ^1^Norwegian Arthroplasty Register, Department of Orthopedic Surgery, Haukeland University Hospital, Bergen; ^2^Department of Orthopedic Surgery, Innlandet Hospital Trust, Elverum; ^3^Department of Surgical Sciences, University of Bergen, Bergen, Norway

## Abstract

**Background and purpose:**

Despite the fact that there have been some reports on poor performance, titanium femoral stems intended for cemented fixation are still used at some centers in Europe. In this population-based registry study, we examined the results of the most frequently used cemented titanium stem in Norway.

**Patients and methods:**

11,876 cases implanted with the cemented Titan stem were identified for the period 1987–2008. Hybrid arthroplasties were excluded, leaving 10,108 cases for this study. Stem survival and the influence of age, sex, stem offset and size, and femoral head size were evaluated using Cox regression analyses. Questionnaires were sent to the hospitals to determine the surgical technique used.

**Results:**

Male sex, high stem offset, and small stem size were found to be risk factors for stem revision, (adjusted RR = 2.5 (1.9–3.4), 3.3 (2.3–4.8), and 2.2 (1.4–3.5), respectively). Patients operated in the period 2001–2008 had an adjusted relative risk (RR) of 4.7 (95% CI: 3.0–7.4) for stem revision due to aseptic stem loosening compared to the period 1996–2000. Changes in broaching technique and cementing technique coincided with deterioration of the results in some hospitals.

**Interpretation:**

The increased use of small stem sizes and high-offset stems could only explain the deterioration of results to a certain degree since the year 2000. The influence of discrete changes in surgical technique over time could not be fully evaluated in this registry study. We suggest that this cemented titanium stem should be abandoned. The results of similar implants should be carefully evaluated.

Femoral stems made of titanium alloys are widely used for total hip arthroplasty (THA), with cementless fixation. Titanium stems for cemented fixation have been discredited after reports on early revisions due to thigh pain, cortical thickening, and femoral osteolysis. The problems have supposedly been caused by crevice corrosion (Willert et al. 1996). There have also been reports on favorable outcome of cemented titanium stems (Acklin et al. 2001, Eingartner et al. 2001, Baumann et al. 2007), and cemented titanium stems are still used at a number of centers. In Norway, a titanium femoral stem intended for cemented fixation has been widely used since 1984. In a recent registry-based study of cemented THA, the performance of this stem (Titan) deteriorated from about the year 2000 (Espehaug et al. 2009). Furthermore, surgeons with extensive experience of the stem have contacted the registry in the last few years with concerns about cases of early loosening. We have therefore studied the performance of this particular stem design in more detail in our population-based arthroplasty register, especially regarding implant size, offset, and any changes in surgical technique including cementing technique.

## Patients and methods

The Norwegian Arthroplasty Register has collected individual data on primary and revision hip arthroplasty procedures since September 15, 1987. The register has been fully described and validated in earlier reports (Havelin et al. 2000, Espehaug et al. 2006). During the period April 1987 to January 1, 2008, 129,481 primary THAs were reported to the register. 11,876 primary THAs with the cemented Titan stem (DePuy, Leeds, UK since 1997; before that, Landanger, La Ciotat, France) were identified in the study period. We excluded hybrid arthroplasties (cemented stem and uncemented cup: 1,768 hips) and we checked that bone cements with known poor results were not used in the cases included (Havelin et al. 1995, Espehaug et al. 2002). Thus, 10,108 hips in 8,938 patients were included in the present study.

**Figure 1.**
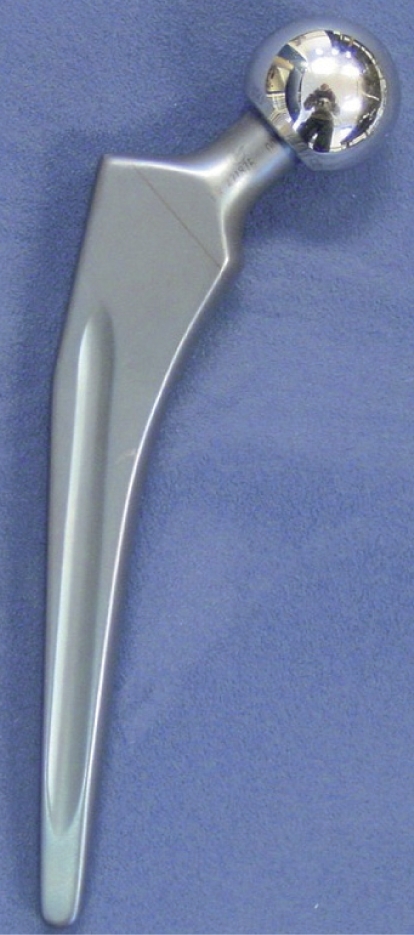
The Titan cemented femoral stem.

The Titan is a straight double-tapered Müller-type stem made of Ti6A14V (Figure 1). The surface is shot-blasted and electrochemically anodized and the surface roughness (Ra) is 0.8 μm. The stem is available in 11 sizes, and the 8 intermediate sizes have a lateralized neck option. According to the manufacturer, the lateralization adds 3.5 mm of femoral offset compared to the standard stems. The offset is also affected by the head/neck length. The CCD angle of the Titan stem is 44.9°; thus, n mm of added head/neck length yields n times cos 44.9º mm of offset. The total offset, consisting of stem type (lateralized/standard) and head/neck length, was calculated in each case, and the hips were stratified into 5 groups according to the total offset. In a concurrent study (unpublished data) we have analyzed retrieved Titan stems; the actual offset differences between lateralized and standard stems were measured on 9 lateralized (L) and 7 standard (S) stems.

We sent a questionnaire to the 10 departments that had used the stem most frequently. The questionnaire included questions on surgical technique and whether the surgical technique had changed with time. Two departments were asked to provide radiographs that they believed to be typical for the revised cases.

### Statistics

Kaplan-Meier survival curves were calculated, and Cox regression analyses were used to evaluate the effect of factors such as age, sex, time of operation (stratified into 3 periods: 1987–1995, 1996–2000, and 2001–2008), stem offset (standard or lateralized), total offset, and stem size (T ≤ 11, T 12–13, T ≥ 14). The main endpoint in the analyses was stem revision for aseptic stem loosening, but we also performed analyses with stem revision for any reason as the endpoint. Survival curves were stopped when less than 100 cases remained at risk. 270 patients were operated on bilaterally and both hips were included in the analyses; the effect of bilateral inclusions in register studies has been found to be negligible (Robertsson and Ranstam 2003, Lie et al. 2004).

## Results

The Titan stem was used throughout the study period. Thus, the follow-up time ranged from 0 to 21 years. The median follow-up time as calculated with the reverse Kaplan-Meier method was 6.3 years (95% CI: 6.1–6.4) (Schemper and Smith 1996). There was no significant difference in age and sex between the 3 time-period groups, whereas the proportion of patients with osteoarthritis as the primary diagnosis increased with time (chi-squared test, p < 0.001) (Table 1). Male sex had a relative risk (RR) of revision that was 2.5 times higher than that for females (for stem revision due to aseptic stem loosening) (95% CI: 1.9–3.4; p < 0.001).

**Table 1.** Patient characteristics and femoral stem epidemiology

	1987–1995	1996–2000	2001–2008
No. of hips	2,895	2,635	4,483
No. of hospitals with > 100 hips in the period	8	8	8
Patient age (median, range)	73.8	74.7	74.7
Sex (% males)	28	26	27
Diagnosis (% osteoarthritis)	70	74	78
Stem size (%)			
T ≤ 11	29	32	39
T = 12–13	45	48	44
T ≥ 14	26	20	17
Stem offset (% lateralized stems)	4.5	5.1	9.4
Femoral head size (%)			
28 mm	26	95	100
32 mm	74	5	0
Femoral head material (%)			
Steel	80	52	0
Cobalt chrome	0	44	95
Others	20	7	4
Bone cement (%)			
Palacos	16	4	0
Palacos with Gentamycin	79	95	61
Refobacin Palacos	0	0	5
Palacos R+G	0	0	17
Refobacin Bone Cement R	0	0	14
Simplex	4	0	0
Others/Missing **^a^**	1	1	3

**^a^**Simplex-Erythromycine/Colistin, Palacos E-Flow, Simplex with Tobramycine, Cemex with Gentamycin, SmartSet HV, Cemex Systeme Genta Fast, Optipac Refobacine Bonecement.

**Figure 2.**
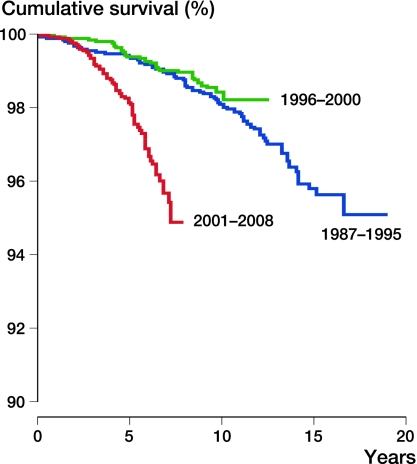
Survival of the stem in stratified time periods. The endpoint was revision of the stem for aseptic loosening.

Survival of the stems that were inserted up to the year 2000 was excellent, with regard to both aseptic stem loosening and stem revision for any reason. In the time period 2001–2008, the results were clearly poorer, and the RR in this period was 4.7 times higher than for the period 1996–2000 (95% CI: 3.0–7.4; p < 0.001). All numbers given from Cox analyses have been adjusted for age, sex, and diagnosis (Table 2 and Figure 2).

**Table 2.** Kaplan-Meier survival and Cox regression analysis with adjustments made for age, sex, and diagnosis. The endpoint was revision of the stem for aseptic loosening

	Relative risk of revision (95% CI)	p-value	Survival at 7 years (95% CI)
1987–1995	1.3 (0.8–2.1)	0.1	99.0 (98.6–99.4)
1996–2000 (ref.)	1	–	99.0 (98.6–99.5)
2001–2008	4.7 (3.0–7.4)	< 0.001	95.7 (94.6–96.8)

**Figure 3.**
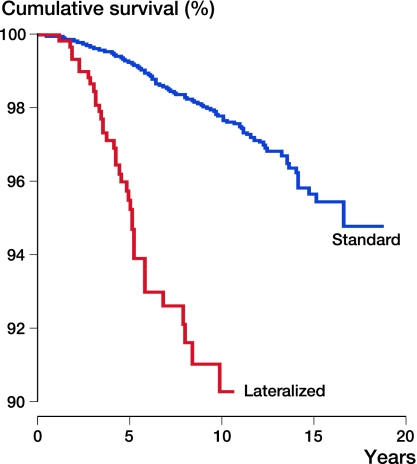
Example of a revision case 3 years after surgery (A), and 5 years after surgery showing extensive osteolysis (B and C). Another case at 2 years (D) and 6 years (E) with a similar appearance.

The failed stems typically presented with extensive osteolysis with or without concomitant stem loosening (Figure 3).

**Figure 4.**
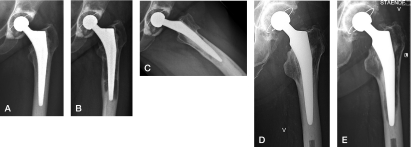
Survival of the stem according to femoral offset (standard vs. lateralized). The endpoint was stem revision for any reason.

The use of stem sizes T11 or smaller, and of lateralized stems increased with time (chi-squared test, p < 0.001) and the most pronounced increase was in the latest period. Cox regression analyses indicated that the lateralized stems had an RR that was 3.3 (95% CI: 2.3–4.8, p < 0.001) times higher than for stem revision of standard stems (Table 3 and Figure 4).

**Table 3.** Cox regression analysis with adjustments for time period and diagnosis. The impact of age, sex, neck option, femoral stem size, and femoral head size. The endpoint was stem revision for aseptic loosening

	Relative risk of revision (95% CI)	p-value
Age	0.9 (0.9–1.0)	< 0.001
Sex **^a^**	2.5 (1.9–3.4)	< 0.001
Neck option: standard (ref.)	1	–
lateralized	3.3 (2.3–4.8)	< 0.00 1
Stem size:		
≤ 11	2.2 (1.4–3.5)	< 0.001
12–13	1.1 (0.7–1.8)	0.7
≥ 14 (ref.)	1	–
Femoral head size:		
32 mm (ref.)	1	–
28 mm	0.9 (0.5–1.6)	0.8

**^a^** Female sex was the reference group (RR = 1).

For the retrieved stems, we found a mean difference of 8.1 mm (range 6.7–9.0) in offset between stem sizes 10 through 14—as opposed to 3.5 mm that was stated in the product marketing literature. The manufacturer later confirmed that the total offset between S and L stems was 7.7 mm across the range, and this was used for the “total offset” calculations. The stems with the highest total offset had 16 mm higher offset than the ones with the lowest offset. The risk of stem revision increased with increasing total offset (Table 4).

**Table 4.** The total offset (stem type and head/neck length) stratified into 5 groups. Relative risk (RR) of stem revision for aseptic stem loosening (Cox, adjusted for sex, age, stem size, and femoral head size)

Total offset	No. of hips	No. of standard stems	No. of lateralized stems	RR (95% CI)
≤ 3 mm	2,969	2,969	0	–
3–5 mm	4,391	4,391	0	1.6 (1.1–2.5)
5–7 mm	1,411	1,411	0	1.4 (0.8–2.4)
7–9 mm	495	238	257	5.1 (3.0–8.9)
> 9 mm	504	86	418	3.9 (2.2–6.9)

**Figure 5.**
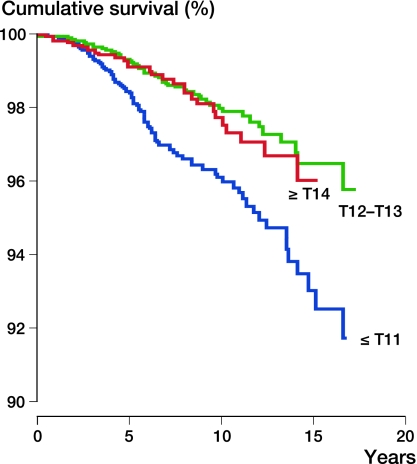
Survival of the stem according to stem size. The endpoint was stem revision for any reason.

Stems of size T11 or smaller were shown to have an RR of revision that was 2.2 times that for stem sizes of T14 or larger (RR = 2.2, CI: 1.4–3.5; p < 0.001) (Figure 5 and Table 3). The poorer results with the smaller stems and the lateralized stems were also found when looking at each time period separately.

However, when the RR (with any stem revision as endpoint) was calculated with adjustments made for all the risk factors mentioned—including stem size and lateralization/total offset—the risk of stem revision was still 3.9 (CI: 2.5–6.3) times higher in the period 2001–2008 than in the period 1996–2000. Thus, the poorer survival in the latest period was only partially caused by increased use of small stems and lateralized stems.

The use of 32 mm femoral heads decreased, and the use of 28 mm heads increased from 1992 through 1998. Head size had no statistically significant effect on survival of the stem (Table 3).

7 of 10 departments responded to the questionnaires. During the inclusion period, there was a change of broach design from sharp, “aggressive” broaches to smoother impacting broaches. The exact year of transition is known for only one department. However, the other departments estimated that the transition took place some time between 1996 and 2002. The company that supplied the broaches could not state the exact year in which the broaches were exchanged at the individual departments. 5 of the 7 departments reported that there had been a trend towards a standard cementing technique since the late 1990s. Prior to this, the “French paradox” cementing technique (meaning that the largest stem possible was inserted with a thin cement mantle) was the technique preferred by most surgeons (Langlais et al. 2003, Skinner et al. 2003). There was no tendency of worse results for hospitals that changed their cementing technique compared to the hospitals that continued to use the “French paradox” technique throughout the inclusion period.

## Discussion

In Norway, 2 types of cemented titanium stems have been used since the national registry started in 1987. Until recently, both were among the best-performing implants in the country. The ITH stem (Smith and Nephew, Memphis, TN) was used until 2005 and had a revision rate of 4.1% at 18 years (Espehaug et al. 2009). The Titan stem also performed excellently until the year 2000. We have shown that small stem size and high stem offset were risk factors for revision, but these factors could only partly explain the shift from excellent results to poor results.

Femoral offset has been found to show a positive correlation with hip stability, range of motion, and abductor muscle strength (McGrory et al. 1995). The negative effects of large offset and small stem size on prostheses survival were recently documented in another registry study (Thien and Karrholm 2010). High offset not only increases the lever arm for abduction, but it also increases the mechanical strain and lever arm on the femoral stem in varus and retroversion. The combination of small stem and high offset creates higher strain on a reduced implant surface area, which could lead to early debonding and eventually loosening (Asayama et al. 2005).

The increased use of high-offset stems and smaller stem sizes in the present study indicates a change in operative technique. There is no reason to believe that the alterations in stem size and offset were caused by any change in the patient population. Patient age and sex were not significantly different in the 3 different time periods. The only difference that emerged was the proportion of osteoarthritis, which had increased in the last period. We do not, however, believe that this change can affect stem size and stem offset to a clinically relevant degree.

Around 1999, the material of the femoral head was changed from stainless steel to cobalt chrome. This change of femoral head material coincided with the deterioration in the results. However, the influence of this transition could not be evaluated in the present study because the 2 materials were used in different time periods. Cobalt chrome has been the material of choice for many years, and there have been no reports to suggest that cobalt chrome gives poorer results than stainless steel. Thus, it is unlikely that the transition to cobalt chrome heads was responsible for the poor results after 2000.

The bone cements used were the same throughout the study period, and they were Palacos-based cements in 97% of cases (Table 1). According to the manufacturers, no modifications were made to these products in terms of chemical composition and manufacturing process during the study period. The Palacos bone cements sold by Biomet and Schering-Plough were, however, removed from the market in 2005 and replaced by the Palacos R+G produced by Heraeus Medical. Since then, Biomet have produced a Palacos copy, the Refobacin Bone Cement. The latter two have been the predominant bone cements in Norway since 2005. All these 4 variants of the Palacos bone cement were used during the study period. There has been some controversy regarding the comparability of the four, and some authors have found differing mechanical and handling properties (Dall et al. 2007, Bridgens et al. 2008).

According to a recent registry study, the four cement brands had equal results when used with the most common cemented hip prostheses in Norway (Espehaug et al. 2009). Results with the Charnley stainless steel stem, which has been the most commonly used stem on a national basis since 1987, actually improved after the year 2000 when used with the same bone cements as the Titan. These findings contradict the hypothesis that changes to the bone cements were responsible for the poorer results since 2000.

Change from manual mixing to vacuum mixing of the bone cement alters the physical properties of the cement (Muller et al. 2002). Specifically, vacuum mixing gives increased shrinking during curing of the cement. This could affect the performance of the stem. Unfortunately, we do not have information on which mixing method was used in the individual cases. We do know, however, that the transition from manual mixing to vacuum mixing occurred in Norwegian hospitals during the years 1990 to 1996. Thus, it is unlikely that the change of mixing method affected the results of the stem in question.

The “French paradox” involves using the same stem size as the largest broach used. This technique produces a thin cement mantle, which is more prone to defects in the mantle. However, good results have been documented using this technique, and in a comparative study the “French paradox” gave outcome that was comparable to that with a standard cementing technique (Skinner et al. 2003). Our register does not include details on the cementing technique used in individual patients. According to the questionnaires we sent out, the use of the “French paradox” technique seemed to lose ground compared to a standard technique over a period of time in the late 1990s. Thus, there was a change in surgical technique that roughly coincided with the deterioration in outcome. However, there was no difference in results between the hospitals that used the “French paradox” throughout the whole inclusion period and the hospitals that changed to a standard cementing technique. The effect of the change in cementing technique is therefore uncertain.

The broaches were modified from sharp cutting broaches to smoother impacting broaches during the inclusion period. We could not, however, find the transition time in the different departments with any degree of accuracy. The registry database does not include details of surgical instrumentation. Thus, the influence of the different broaches on implant survival cannot be evaluated. The characteristics of the bone bed influence the quality of cementation, and eventually the clinical results. Sharp broaches remove some of the spongeous bone whereas smooth broaches impact this devitalised bone. Theoretically, intrusion of bone cement into an impacted bone bed would be poorer than into a more porous bone bed. This, in turn, could alter the quality of primary fixation. Furthermore, when bone is impacted rather than removed, stability of the broach may be achieved with a smaller broach thus dictating a smaller stem to be implanted. The change of broaches and the increased use of smaller stems coincided, but this finding does not prove a causative relationship. According to the manufacturer of the stem, no modifications were made to the implant.

The information from the manufacturer on the offset differences between standard and lateralized stems was inconsistent, and our findings have later been confirmed by the manufacturer. Inaccurate data from a manufacturer of implants for clinical use is of course inconvenient.

Whether the current problems with this stem are due to alterations in surgical technique, instrumentation, or the characteristics of bone cement cannot be determined from this study. The mechanical properties of titanium may mean less tolerance to minute changes in the surgical technique, especially the cementation, than less elastic materials such as steel or cobalt chromium. We are currently analyzing retrieved Titan stems to search for any differences in the surface or geometry of the stems from the last time period compared to earlier stems, with a view to investigating the mechanism(s) of failure.

Despite the fact that there have been some positive reports on cemented titanium stems (Acklin et al. 2001, Baumann et al. 2007, Eingartner et al. 2001), our findings and the accumulated negative results with these implants suggest that they should be abandoned.
